# Supplementary material for: Functional Selectivity of Coumarin Derivates Acting via GPR55 in Neuroinflammation
Source: Int J Mol Sci. 2022 Jan 16;23(2):959. doi: 10.3390/ijms23020959 (PMC8779649; doi:10.3390/ijms23020959)
Supplement: Supplementary file 1 [file ijms-23-00959-s001.zip › ijms-1548166-supplementary.pdf]

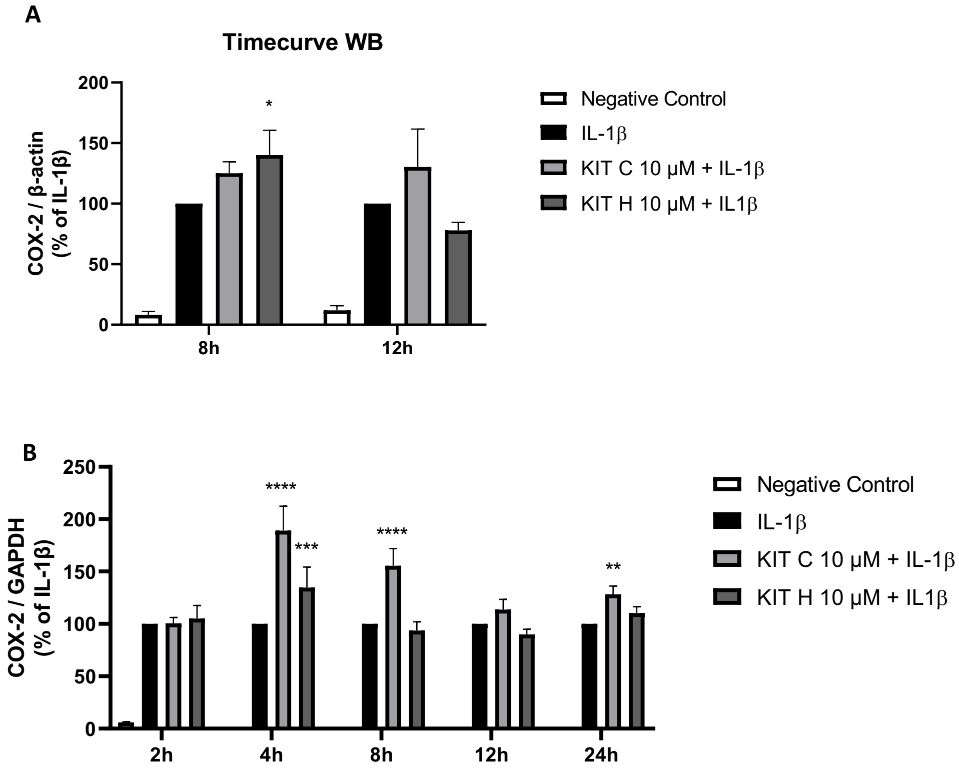

Figure S1: Time dynamic effects of KIT C (light grey bars) and KIT H (dark grey bars) on COX-2 protein (A) and mRNA (B) levels in IL-1β-stimulated SK-N-SH. Cells were treated as described under material and methods. Values are presented as the mean ± SEM of at least three independent experiments. Statistical analysis was performed using one-way ANOVA with Dunett's post hoc tests (A) or two-way ANOVA with following post hoc tests with *$p < 0.05$, **$p < 0.01$, ***$p < 0.001$, ****$p < 0.0001$ compared to IL-1β-stimulated cells.
